# Associations between retinol-binding protein 4 and cardiometabolic risk factors and subclinical atherosclerosis in recently postmenopausal women: cross-sectional analyses from the KEEPS study

**DOI:** 10.1186/1475-2840-11-52

**Published:** 2012-05-15

**Authors:** Gary Huang, Dan Wang, Unab I Khan, Irfan Zeb, JoAnn E Manson, Virginia Miller, Howard N Hodis, Matthew J Budoff, George R Merriam, Mitchell S Harman, Eliot A Brinton, Marcelle I Cedars, Yali Su, Rogerio A Lobo, Frederick Naftolin, Nanette Santoro, Hugh S Taylor, Rachel P Wildman

**Affiliations:** 1 Department of Epidemiology & Population Health, Albert Einstein College of Medicine, Bronx, NY, USA; 2 Department of Pediatrics, Albert Einstein College of Medicine, Bronx, NY, USA; 3 Division of Cardiology, Los Angeles Biomedical Research Institute at Harbor-UCLA, Torrance, CA, USA; 4 Department of Medicine, Brigham and Women’s Hospital, Harvard Medical School, Boston, MA, USA; 5 Departments of Surgery and Physiology and Biomedical Engineering, Mayo Clinic, Rochester, MN, USA; 6 Atherosclerosis Research Unit, University of Southern California, Keck School of Medicine, Los Angeles, CA, USA; 7 VA Puget Sound Health Care System and Department of Medicine, University of Washington School of Medicine, Seattle, WA, USA; 8 Kronos Longevity Research Institute, Phoenix, AZ, USA; 9 Department of Cardiovascular Genetics, University of Utah School of Medicine, Salt Lake City, UT, USA; 10 Department of Obstetrics, Gynecology, and Reproductive Sciences, University of California at San Francisco, San Francisco, CA, USA; 11 SliverCreek Technologies, Gilbert, AZ, USA; 12 Department of Obstetrics and Gynecology, Columbia University College of Medicine, New York, NY, USA; 13 Department of Obstetrics and Gynecology, New York University School of Medicine, New York, NY, USA; 14 Department of Obstetrics and Gynecology and Women’s Health, University of Colorado-Denver School of Medicine, Aurora, CO, USA; 15 Department of Obstetrics, Gynecology, and Reproductive Sciences, Yale School of Medicine, New Haven, CT, USA; 16 Epidemiology & Population Health, Albert Einstein College of Medicine, 1300 Morris Park Avenue, Belfer Building, Room 1309, Bronx, NY 10461, USA

**Keywords:** Retinol-binding protein 4, Subclinical atherosclerosis, Risk factors, Women

## Abstract

**Background:**

The published literature regarding the relationships between retinol-binding protein 4 (RBP4) and cardiometabolic risk factors and subclinical atherosclerosis is conflicting, likely due, in part, to limitations of frequently used RBP4 assays. Prior large studies have not utilized the gold-standard western blot analysis of RBP4 levels.

**Methods:**

Full-length serum RBP4 levels were measured by western blot in 709 postmenopausal women screened for the Kronos Early Estrogen Prevention Study. Cross-sectional analyses related RBP4 levels to cardiometabolic risk factors, carotid artery intima-media thickness (CIMT), and coronary artery calcification (CAC).

**Results:**

The mean age of women was 52.9 (± 2.6) years, and the median RBP4 level was 49.0 (interquartile range 36.9-61.5) μg/mL. Higher RBP4 levels were weakly associated with higher triglycerides (age, race, and smoking-adjusted partial Spearman correlation coefficient = 0.10; *P* = 0.01), but were unrelated to blood pressure, cholesterol, C-reactive protein, glucose, insulin, and CIMT levels (all partial Spearman correlation coefficients ≤0.06, *P* > 0.05). Results suggested a curvilinear association between RBP4 levels and CAC, with women in the bottom and upper quartiles of RBP4 having higher odds of CAC (odds ratio [95% confidence interval] 2.10 [1.07-4.09], 2.00 [1.02-3.92], 1.64 [0.82-3.27] for the 1^st^, 3^rd^, and 4^th^ RBP4 quartiles vs. the 2^nd^ quartile). However, a squared RBP4 term in regression modeling was non-significant (*P* = 0.10).

**Conclusions:**

In these healthy, recently postmenopausal women, higher RBP4 levels were weakly associated with elevations in triglycerides and with CAC, but not with other risk factors or CIMT. These data using the gold standard of RBP4 methodology only weakly support the possibility that perturbations in RBP4 homeostasis may be an additional risk factor for subclinical coronary atherosclerosis.

**Trial registration:**

ClinicalTrials.gov number NCT00154180

## Background

Obesity, a worldwide epidemic, is associated with a dramatic increase in the prevalence of type 2 diabetes, and has been associated with cardiovascular disease (CVD) morbidity and mortality [1-3]. Retinol-binding protein 4 (RBP4), which is released primarily by the liver, has recently been shown to be released by adipose tissue as well [4]. RBP4 down-regulates the glucose transporter GLUT4 [5-7], which acts as the rate-limiting step in insulin-activated glucose transport across both muscle and adipocyte membranes [6]. In humans, circulating RBP4 levels have been shown to be negatively correlated with levels of insulin sensitivity, and to be higher among individuals with obesity and type 2 diabetes, as well as among those with adverse CVD risk factor profiles or metabolic syndrome, frequently independent of body size [6-14]. However, conflicting data also exist [9,15-17]. Discrepancies in published RBP4 data may relate to complications of the RBP4 assay methodology [18], which this study has striven to overcome with western blot analysis.

The purpose of the current study was to examine the associations of RBP4 with both standard CVD risk factors as well as subclinical atherosclerosis in healthy mid-life women screened for the Kronos Early Estrogen Prevention Study (KEEPS). Western blot, the gold standard analytic technique for RBP4 [18], was used for RBP4 analysis to overcome problems associated with other analytical techniques.

## Methods

### Study population

The current cross-sectional analyses utilize data from the KEEPS, a randomized, placebo-controlled, double-blinded, prospective trial of the effects of menopausal hormone therapy on the progression of subclinical atherosclerosis in recently menopausal women. KEEPS is a multi-center trial with 9 centers (University of Utah, University of California at San Francisco; Brigham and Women’s Hospital; Mayo Clinic, Rochester; Columbia University College of Physicians and Surgeons;VA/ University of Washington; Yale University; Montefiore Medical Center; Kronos Longevity Research Institute). KEEPS inclusion criteria were age 42–58 years, at least 6 months but no more than 36 months of amenorrhea and last spontaneous menses occurring after age 40, FSH values ≥35 ng/ml and/or estradiol levels <35 pg/ml, and good general health. Women reporting use of estrogen- or progestin-containing medication (oral contraceptive or hormone replacement), selective estrogen receptor modulators (SERMs), or medications with significant estrogenic activity, such as isoflavones, in the prior 3 months, a history of clinical CVD, current heavy smoking (greater than one half pack per day), a body mass index (BMI) ≥35 kg/m^2^, LDL cholesterol > 190 mg/dL or triglycerides >400 mg/dL, diabetes, uncontrolled hypertension, or with moderate subclinical CVD, defined as a coronary artery calcium (CAC) score ≥50 Agatston units, were ineligible for randomization. Women meeting eligibility criteria were randomized to one of three treatment groups: oral conjugated equine estrogen (Premarin, 0.45 mg/day) with oral micronized progesterone (12 days per month), transdermal 17β-estradiol (via skin patch, Climara, 50 μg/day) with oral micronized progesterone (12 days per month), and one daily placebo group (inactive pill/patch) with placebo micronized progesterone (12 days per month). The current analyses utilize data from the screening and baseline KEEPS visit, prior to randomization and receipt of any estrogen therapy or placebo. A total of 1,046 women were screened between July 2005 to June 2008, with 775 of them passing earlier screening components and eventually receiving a CT scan for the purposes of confirming CAC Agatston score eligibility. Of these, 66 women were missing RBP4 data, leaving 709 women for the current analyses. The institutional review boards of the participating institutions approved this study, and all women signed informed consent.

### Blood measures

All blood specimens were collected following an overnight fast, then frozen at −70°C on site until they were either processed locally, or sent to the Kronos Science Laboratory (Phoenix, AZ, USA) for storage or assays. Complete blood count and chemistry panel were performed at the clinical laboratories at each recruiting center. C-reactive protein (CRP) and insulin were assayed by DPC Immulite 2000 (Diagnostic Products Corporation, Los Angeles, CA).

RBP4 in human serum was measured by quantitative western blotting [18]. Briefly, native RBP4 purified from human serum (Hytest) was used for assay calibration. Each gel (18 wells) contained a molecular weight marker, five concentrations of RBP4 standards, four low and high levels of controls made by human serum, and eight patient samples. RBP4 standards were placed in the same position at the center of each gel. RBP4 standard solutions of 15, 30, 60, 120, and 240 μg/ml were prepared in 10 mM phosphate buffer with 150 mM NaCl (pH 7.4). Standards, controls, and patient samples were diluted 1:200 in SDS-PAGE buffer and boiled for 5 min. 25 μl of diluted standards, controls, patient samples, and a molecular weight marker (Bio-Rad precision plus protein standards kaleidoscope) were then electrophoresed on 18% Criterion Tris-glycine SDS-PAGE gels ( BioRad) and transferred to nitrocellulose. Western blotting was performed with 5% non-fat dry milk in tris-buffered saline with 1% of detergent as a blocking agent. Blots were incubated overnight at 4°C with rabbit anti-human RBP primary antibody (DAKO) diluted 1:1,000 and for 1 h at room temperature with ECL horseradish-peroxidase-conjugated secondary antibody (Fisher) diluted 1:2,000. Bands were detected by enzymatic chemiluminescence and quantified using the Kodak Gel Logic 100 Digital Imaging System and Carestream Molecular Imaging Software. A single band for RBP4 migrated at about 21 kDa. Exponential curves were used to fit the RBP4 standards for each individual gel and calculate concentrations in patient samples. The gel to gel variation (inter precision) was monitored by two levels of controls with CV less than 15%. The control results of each gel were reviewed to ensure the assay quality. If the control results were outside of the acceptable limits determined by the assay method validation, the run was rejected, and all samples on the same gel were repeated.

### Anthropometric and lifestyle exposures

Baseline smoking status (current use yes/no) and race-ethnicity were obtained through self-reported questionnaire. Height (cm) and weight (kg) of participants, wearing light clothing and without shoes, was measured with stadiometers and calibrated balance-beam scales, respectively. BMI was calculated as weight in kilograms divided by the square of height in meters. Waist circumference (cm) was measured with a non-stretchable tape at the end of a normal expiration, at the smallest horizontal circumference between the ribs and iliac crest. Blood pressure was measured in the right arm in the seated position, after at least a 5 minute rest, and was averaged across two readings.

### Subclinical atherosclerosis measures

Carotid artery intima-media thickness (CIMT) was measured from high resolution B-mode ultrasound images obtained with a 7.5 MHz transducer at both the screening and baseline KEEPS visits. Carotid artery images were acquired at each study center by certified ultrasound technicians trained at the core CIMT imaging and reading center (PI: Dr. Howard Hodis, USC) to perform standardized image acquisition. Image acquisition was optimized for minimal measurement variability (coefficient of variation <2%) using methodology developed specifically for reproducible longitudinal imaging employing internal anatomical structural probe angulation techniques including vessel stacking, baseline image matching, electrocardiographic gating, and individualized instrumentation conditions (Patents 2005, 2006, 2011) [19]. All images were analyzed by an experienced investigator at the core CIMT imaging and reading center using in-house developed computerized automated edge detection software employing subpixel interpolation (Patents 2005, 2006, 2011) [19]. CIMT values used in the current analyses represent an average of the screening and baseline CIMT measurements.

Each participant underwent non-enhanced cardiac computed tomography (CT) scans for assessment of coronary atherosclerosis. Scans were acquired either via electron beam CT (GE/Imatron, Inc.), or via multidetector CT using either a General Electric helical scanner or a Siemens or Phillips multi-slice scanner (minimum requirement 4 detector heads). Comparability among centers was assured by regular calibration using a standard phantom. The scans were obtained during a single breath hold at end inspiration to reduce motion artifacts and improve image quality of the coronary arteries. Coronary calcium was defined as a plaque of at least 3 contiguous pixels (area 1.02 mm^2^) with a density of >130 Hounsfield units. The individual lesion score was calculated by multiplying the lesion area by a density factor derived from the maximal Hounsfield unit within this area, as described by Agatston et al. [20]. A total CAC score was determined by summing the individual lesion scores from each of 4 anatomic sites (left main, left anterior descending, circumflex, and right coronary). A single experienced investigator, blinded to the subject identity, interpreted all the scans using commercially available software (TeraRecon, Foster City, CA).

### Statistical analyses

Mean (standard deviation), median (interquartile range), or n (%) were tabulated for demographic, behavioral, and laboratory characteristics by quartiles of RBP4. Differences in the distributions of these variables across RBP4 quartiles were calculated by the Chi-Square test for categorical variables, ANOVA for normally distributed continuous variables, and the Kruskal-Wallis test for non-normally distributed continuous variables. Following this, partial Spearman correlation coefficients were calculated for associations between RBP4 and cardiometabolic risk factors and CIMT, adjusted for age, race (white vs. non-white), and current smoking. To assess associations between RBP4 and CAC, multivariable adjusted logistic regression was used to calculate odds ratios of CAC score >0 across quartiles of RBP4, as well as treating RBP4 as a continuous variable after log-transformation, adjusted for age, race, and current smoking, and again after additional adjustment for triglycerides. To ensure that residual confounding by smoking was not influencing associations, sensitivity analyses were carried out excluding current smokers from regression analyses.

## Results

**Figure 1:**
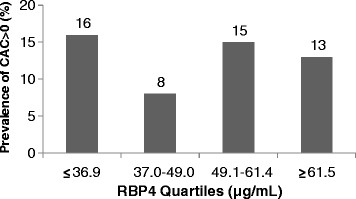
Prevalence (%) of CAC score > 0 by RBP4 Quartiles.

The mean age of study participants was 52.9 (± 2.6) years. Demographic and laboratory characteristics stratified by quartiles of RBP4 are presented in Table1. The mean age, prevalence of current smoking, proportion of non-White participants, and levels of cardiometabolic risk factors did not differ across quartiles of RBP4. With the exception of triglycerides (r = 0.10, *P* = 0.01), the associations of cardiometabolic risk factors and RBP4 remained null (all *P* > 0.05) after multivariable adjustment for age, race-ethnicity, and smoking status (Table2). CIMT was similarly not associated with RBP4 either prior to or after multivariable adjustment (Tables 1 and 2). The prevalence of CAC score>0 was similar in the 1^st^, 3^rd^, and 4^th^ quartiles of RBP4, but appeared lower in the 2^nd^ tertile (Figure1), though this difference was not statistically significant (*P*-value for chi-square test = 0.15). To further explore the possibility of a curvilinear association, multivariable-adjusted logistic regression models for CAC were initially performed with the 1^st^ RBP4 quartile as the reference category (Table3, Model 1), but were repeated utilizing the 2^nd^ RBP4 quartile as the reference category (Table3, Model 2). When using the 1^st^ RBP4 quartile as the reference, there was a statistically significant 51% lower odds of CAC with RBP4 levels in the range of 37 to 49 mg/mL in comparison to the lowest RBP4 levels (≤36.9 mg/mL) after adjustment for age, race, and current smoking (Table3, Model 1). However, in comparison to the first quartile, the 3^rd^ and 4^th^ RBP4 quartiles were not significantly associated with CAC. When using the 2^nd^ RBP4 quartile as the reference category (37.0-49.0 mg/mL) (Table3, Model 2), a curvilinear relationship between RBP4 quartiles and CAC was suggested, with both lower and higher RBP4 quartiles associated with higher odds of CAC. However, despite this pattern, a squared RBP4 term tested in the context of logistic regression was not statistically significant (p = 0.10). There was no evidence of a linear association between RBP4 and the prevalence of CAC score >0 (Table3, Model 3). Given the statistically significant association between triglycerides and RBP4, CAC analyses were additionally adjusted for triglycerides. The magnitude of odds ratios was very similar to models without inclusion of triglycerides (ORs [95% CIs]: 2.11 [1.08-4.11], 1.93 [0.99-3.79], 1.59 [0.79-3.16] for the 1^st^, 3^rd^, and 4^th^ RBP4 quartiles, respectively, vs. the 2^nd^, adjusted for age, race, current smoking, and triglycerides). Due to the curvilinearity suggested by the RBP4-CAC relationship, logistic regression analyses tabulating odds ratios of the top tertile of each cardiometabolic risk factor associated with being in the 2^nd^ RBP4 quartile vs. the 1^st^, 3^rd^, and 4^th^ were carried out similar to Model 2 in Table3. Curvilinearity was not suggested for any of the cardiometabolic risk factors examined or CIMT. Sensitivity analyses excluding current smokers from regression models resulted in nearly identical odds ratios and 95% confidence intervals to those including current smokers.

**Table 1:** Study Characteristics by Quartiles of RBP4

	**RBP4 Quartile, μg/mL**				
	**≤36.9**	**37.0-49.0**	**49.1-61.4**	**≥61.5**	*P*-value**
Age, years	52.7 (0.2)	53.1 (0.2)	52.7 (0.2)	53.1 (0.2)	0.33
% Current Smokers	8.0%	6.7%	6.8%	4.5%	0.21
% Non-White Race	29.6%	21.9%	21.5%	21.4%	0.08
BMI, kg/m^2^	26.1 (0.3)	26.2 (0.3)	26.3 (0.3)	26.4 (0.3)	0.49
Waist Circumference, cm*	82.0 (16.5)	82.7 (15.2)	83.8 (18.0)	85.8 (17.0)	0.42
Systolic Blood Pressure, mmHg	118.1 (1.1)	119.4 (1.1)	117.2 (1.1)	120.3 (1.2)	0.38
Diastolic Blood Pressure, mmHg	74.7 (0.7)	75.3 (0.7)	73.8 (0.6)	75.6 (0.7)	0.66
HDL-C, mmol/L	1.71 (0.03)	1.68 (0.03)	1.66 (0.04)	1.71 (0.04)	0.94
Triglycerides, mmol/L*	0.82 (0.53)	0.86 (0.53)	0.94 (0.53)	0.92 (0.57)	0.09
Glucose, mmol/L	4.95 (0.04)	4.93 (0.04)	4.96 (0.04)	4.94 (0.04)	0.94
Insulin, pmol/L*	27.8 (31.9)	27.8 (36.1)	28.5 (34.7)	34.0 (43.8)	0.23
C-reactive Protein, mg/dL*	0.8 (1.9)	1.2 (2.2)	1.1 (2.1)	0.9 (2.2)	0.14
Carotid IMT, mm*	0.71 (0.11)	0.70 (0.11)	0.72 (0.10)	0.72 (0.11)	0.52

Values are mean (standard deviation) unless otherwise indicated. *median (interquartile range).

**P-value from Chi-Square test for categorical variables, ANOVA for normally distributed variables, and from the Kruskal-Wallis Test for non-normally distributed variables.

*RBP4* retinol-binding protein 4; *BMI* body mass index; *IMT* intima-media thickness.

**Table 2:** Partial* Spearman Correlation Coefficients for Associations between RBP4 and Cardiometabolic Factors and Carotid IMT

	**BMI**	**Waist Circum.**	**HDL-C**	**Triglycerides**	**Glucose**	**Insulin**	**C-Reactive Protein**	**Carotid IMT**
**r**	0.02	0.04	−0.0006	0.10	0.002	0.06	0.03	0.03
**p-value**	0.55	0.26	0.99	**0.01**	0.97	0.10	0.50	0.37

*Adjusted for age, race-ethnicity, and current smoking.

*RBP4* retinol-binding protein 4; *BMI* body mass index; *IMT* intima-media thickness.

**Table 3:** Adjusted* Odds Ratios of CAC > 0 associated with RBP4

	**Odds Ratio**	**95% CI**
**Model 1**		
RBP4 Quartiles (μg/mL)		
≤36.9 (Reference)	1.00	----
37.0-49.0	0.49	0.25-0.95
49.1-61.4	0.95	0.54-1.69
≥61.5	0.78	0.43-1.42
**Model 2**		
RBP4 Quartiles (μg/mL)		
≤36.9	2.10	1.07-4.09
37.0-49.0 (Reference)	1.00	----
49.1-61.4	2.00	1.02-3.92
≥61.5	1.64	0.82-3.27
**Model 3**		
Per μg/mL increase in log-RBP4	1.00	0.55-1.81

*All three models adjusted for age, race-ethnicity, and current smoking.

*RBP4* retinol-binding protein 4.

## Discussion

Among these healthy, recently postmenopausal women, higher RBP4 levels were weakly associated with greater triglyceride levels, and both low and high RBP4 levels were associated with approximately twice the odds of the presence of CAC. No associations were observed between RBP4 levels and other cardiometabolic risk factors or CIMT.

RBP4 has long-been known to be released by the liver, but recently, it has been shown that approximately 15% of circulating RBP4 levels results from adipose tissue RBP4 secretion [6]. RBP4 down-regulates GLUT4 [6,7], the insulin-activated glucose transporter responsible for translocation of glucose into both muscle and fat cells [6], and has also recently been shown to induce expression and secretion of pro-inflammatory cytokines in primary human macrophages known to induce insulin resistance [21]. In humans, circulating RBP4 levels were found to be highly negatively correlated with levels of insulin sensitivity, and to be increased with obesity and in those with type 2 diabetes [6-14]. However, discrepant results from other studies have questioned these associations, failing to demonstrate associations with either obesity or indicators of glucose homeostasis [9,15-17]. Results from the present study also found no association between RBP4 and BMI, waist circumference, glucose, or insulin levels. In addition to possible measurement error resulting from use of immunoassay in prior studies [18], a potential contributing factor to the discrepant literature is that the relationship between RBP4 and insulin resistance may be stronger in younger individuals than older [22], possibly from changes in body fat distribution with age.

Retinoids have been known to affect the expression of several genes involved in triglyceride metabolism, including regulators of apo C-III production, hepatic and gastrointestinal triglyceride production and secretion, and beta-oxidation of fatty acids [23]. As such, similar to our finding of higher triglyceride levels with higher RBP4 values, prior studies have shown that RBP4 is correlated with serum triglycerides [13,14,22,24-27]. In addition, although prior studies have often documented associations between RBP4 and other cardiometabolic risk factors in addition to triglycerides, some studies support our findings of an association with triglycerides, only. In a cross-sectional analysis of 308 non-diabetic patients with hypertension, Shim et al. discovered associations between RBP4 and indices of metabolic syndrome to be null, with the exception of triglycerides, which yielded a significant positive correlation (r = 0.25, *P* < 0.001) [28]. A study of 473 subjects with normal glucose tolerance revealed similar results, with RBP4 positively correlated with triglycerides (r = 0.10, *P* < 0.001) but not with other cardiometabolic risk factors [29]. The present study extends these findings by demonstrating an association between RBP4 and triglycerides, but not other cardiometabolic risk factors, in a population of healthy, midlife women, utilizing the gold standard RBP4 measurement methodology.

The association between RBP4 and CIMT, a measure of systemic atherosclerosis, has been investigated in only a few studies, and as with relationships between RBP4 levels and obesity, insulin resistance, and other CVD risk markers, results conflict. Despite recent data suggesting that increases in fasting plasma glucose even within the normal range are associated with worse arterial stiffness levels [30], reported results suggest not only positive associations between RBP4, which down-regulates the glucose transporter GLUT-4, and CIMT, but also inverse associations, and similar to our results, null associations [14,31-34]. Null associations with RBP4 as we report here in healthy midlife women have also been reported in type 2 diabetes patients, both for CIMT and for other measures of peripheral subclinical disease including ankle-brachial index and carotid/femoral echography [14,34].

In contrast to our findings with CIMT and to earlier findings noted above with ankle-brachial index, we observed a significantly higher odds of the presence of CAC in women with both low and high RBP4 levels compared to women with moderate (2^nd^ RBP4 quartile) RBP4 levels. The odds of having a CAC score > 0 in women in either the 1^st^ or 3^rd^ quartiles of RBP4 were approximately 2 times higher than that among women with RBP4 levels in the 2^nd^ quartile. Similarly, women in the 4^th^ RBP4 quartile had a non-significantly 64% higher odds of having a CAC score > 0 compared to women in the 2^nd^ RBP4 quartile. To our knowledge, this is the first investigation to assess the association of RBP4 with the presence of CAC. These findings require replication in future studies and need to be interpreted with considerable caution given the lack of a significant squared RBP4 term in regression analyses. Caution is especially indicated given the relatively small number of women with CAC score > 0 in the current study (n = 93 overall, and n = 15 in the 2^nd^ RBP4 quartile, the quartile which appeared to have a lower CAC prevalence), and the null association with CIMT, an alternative measure of atherosclerosis. Had RBP4 levels been associated with measures of glucose homeostasis as in certain prior studies, a u-shaped association with coronary atherosclerosis might be supported given the heightened CVD death not only with hyperglycemia, but also potentially with hypoglycemia, as suggested by the Action to Control Cardiovascular Risk in Diabetes (ACCORD) trial results [35]. However, in the current study, RBP4 levels were not associated with glucose, insulin, BMI, or waist circumference. Should our CAC findings be replicated in a future study, they would be supported not only by one published study finding lower median RBP4 levels in patients with CAD (27.8 μg/mL) vs. controls (29.8 μg/mL),[36] but also by published data suggesting differential associations of CAC vs. CIMT with CVD risk factors and CVD events [37,38].

Some limitations of this study must be taken into consideration. The design of this study was cross-sectional, so the results do not imply causality. Our study sample also included only healthy, recently menopausal women. Therefore, our results may not be applicable to an older population with greater prevalence of atherosclerosis. Additionally, glucose and lipids were obtained from blood collected during screening, while insulin, CRP and RBP4 were obtained from blood collected at the baseline randomization examination. Therefore, we were unable to calculate insulin resistance via the homeostasis model assessment (HOMA-IR), and a bias towards the null was introduced for associations between RBP4 and lipids and glucose. However, insulin levels, despite being measured at the same alternate visit as RBP4, showed moderate to strong associations with lipids and glucose (Spearman correlation coefficient of −0.4 with HDL, 0.4 with triglycerides, and 0.3 with glucose; all *P* < 0.001) but a null association with RBP4 (Spearman correlation coefficient = 0.06; *P* = 0.10), suggesting that null associations between RBP4 and HDL and glucose were not a result of the measurement schedule.

The primary strength of this study involves the method of RBP4 detection. The quantitative western blot methodology that we have chosen has been cited as the gold-standard for RBP4 determination [18], and utilizes the full-length RBP4 protein and monoclonal antibody specific for full-length RBP4 protein, greatly increasing validity compared to numerous prior investigations utilizing immunoassay. This method was employed despite the large sample size of the KEEPS trial, making this the largest investigation to utilize quantitative western blot methodology for assessment of these relationships. In addition, the health of the KEEPS participants enabled assessment of the associations between RBP4 and CVD-related parameters prior to disease expression.

## Conclusions

These analyses in healthy, recently postmenopausal women suggest that higher RBP4 levels are weakly associated with elevations in triglycerides, and that both low and high RBP4 levels may be associated with subclinical coronary atherosclerosis. Given the caution with which the coronary atherosclerosis results should be viewed, our current results in healthy midlife women only weakly support a role for RBP4 homeostasis in cardiometabolic abnormalities and cardiovascular disease. However, future studies should attempt to replicate our CAC findings. If successful, RBP4 may end up warranting examination as a potential target of interventions to reduce CVD risk.

## Abbreviations

ACCORD: Action to control cardiovascular risk in diabetes; BMI: Body mass index; CAC: Coronary artery calcification; CIMT: Carotid intima media thickness; CRP: C-reactive protein; CT: Computed tomography; CV: Coefficient of variation; CVD: Cardiovascular disease; GLUT4: Glucose transporter type 4; KEEPS: Kronos Early Estrogen Prevention Study; LDL: Low-density lipoprotein.

## Competing interests

The authors have no competing interests to disclose.

## Authors’ contributions

GH wrote the manuscript; RPW conceptualized the research hypothesis and analyses, assisted in writing the manuscript, and edited the manuscript; DW performed all of the statistical analyses; JEM and NS assisted in conceptualizing the research question and reviewed and edited the manuscript, MJB, HNH, MSH, and YS researched data, and reviewed and edited the manuscript, EAB, MIC, UIK, RAL, GRM, VM, FN, HST, and IZ reviewed and edited the manuscript. All authors read and approved the final manuscript.
